# Empirical analysis of a plan‐of‐the‐day strategy to approximate daily online reoptimization for prostate CBCT‐guided adaptive radiotherapy

**DOI:** 10.1002/acm2.14221

**Published:** 2023-11-29

**Authors:** Adam D. Yock, Annie Cooney, Manuel Morales‐Paliza, Eric Shinohara, Kenneth Homann

**Affiliations:** ^1^ Department of Radiation Oncology Vanderbilt University Medical Center Nashville Tennessee USA

**Keywords:** adaptive radiotherapy, ART, CBCT‐guided ART, online ART, plan‐of‐the‐day

## Abstract

**Purpose:**

Adaptive radiotherapy (ART) can improve the dose delivered to the patient in the presence of anatomic variations. However, the required time, effort, and clinical resources are intensive. This work analyzed a plan‐of‐the‐day (POD) approach on clinical patients treated with online ART to explore implementations that balance dosimetric benefit and clinical resource cost.

**Methods:**

Eight patients treated to the prostate and proximal seminal vesicles with 26 fractions of CBCT‐guided, daily online ART were retrospectively analyzed. With a plan library composed of daily adaptive plans from the initial week of treatment and the original plan, the effect of a POD approach starting the following week was investigated by simulating use of these previously generated plans under 3‐ and 6‐degree‐of‐freedom patient alignment. The plan selected for each treatment was that from the library that maximized the Dice similarity coefficient of the clinical target volume with that of the current treatment fraction. The resulting distribution of several target coverage and organ‐at‐risk dose metrics are described relative to those achieved with the daily online reoptimized adaptive technique.

**Results:**

The values of target coverage and organ‐at‐risk dose metrics varied across patients and metrics. The POD schemas closely approximated the reference values from a fully reoptimized adaptive plan yet required less than 20% of the reoptimization effort. The POD schemas also had a much greater effect on target coverage metrics than 6‐degree‐of‐freedom registration did. Organ‐at‐risk dose metrics also varied considerably across patients but did not exhibit a consistent dependence on the particular schema.

**Conclusions:**

POD schemas were able to achieve the vast majority of the dosimetric benefit of daily online ART with a small fraction of the online reoptimization effort. Strategies like this might allow for more practical and strategic implementation of ART so as to benefit a greater number of patients.

## 1 INTRODUCTION

Recently, the daily delivery of online adaptive radiotherapy (ART) has been made possible by technological advancements such as treatment units integrated with MRIs as well as linear accelerators featuring AI‐driven, CBCT‐guided replanning software.^1^, ^2^, ^3^, ^4^, ^5^, ^6^, ^7^, ^8^, ^9^, ^10^ These systems optimize new radiotherapy plans each day based on volumetric imaging to account for changes due to patient setup variation and internal anatomic motion. By better responding to these daily variations, online ART has shown the promise of improving the dose delivered to the patient.^4^, ^10^, ^11^, ^12^, ^13^, ^14^, ^15^, ^16^

Although these technologies facilitate a replanning effort that can be accomplished within a reasonable daily treatment appointment duration (e.g., 15−30 min),^5^, ^6^, ^7^, ^9^, ^12^, ^13^, ^14^, ^17^, ^18^, ^19^, ^20^ considerations regarding their practical implementation linger and have implications for the clinical resources required to deliver the treatment safely and effectively—resources such as machine time and the time and effort required of the clinical team. As a new plan is created at the treatment machine while the patient remains in position, each planning task must be completed onsite and in near real‐time. If members of the clinical care team, including the radiation therapist, dosimetrist, physicist, and radiation oncologist, maintain their conventional responsibilities during the adaptive workflow, their presence will be required at the treatment machine whenever online ART is being considered. This represents a novel demand of their time that competes with their non‐adaptive tasks and workload, and which has the potential to directly and indirectly affect clinical operations throughout the radiotherapy department. In addition, time and effort beyond that required for an individual to complete their own specific tasks may be necessary due to the particular adaptive workflow and to changing and often unpredictable clinical schedules. Several works have tracked the clinical resources required for various ART workflows in terms of time and monetary cost.^20^, ^21^, ^22^ These effects can make the implementation of daily online ART in a conventional radiotherapy department particularly disruptive, and the utilization of the technology must be considered strategically. While, in principle, all patients could benefit dosimetrically by delivering a daily reoptimized treatment plan, implementation on that scale remains impractical without significant changes in departmental staffing, structure, or policies. Furthermore, indiscriminate or poorly selected utilization of daily online ART—for example, for a patient who does not experience large changes in daily anatomy—may obscure its true clinical impact and discourage its proper use for patients that are likely to benefit.

The strategic implementation of online ART can be informed by considering the anatomic differences the technique can address in relation to other techniques that are also available to address them. Consider, for example, the following relationships between two anatomic configurations. Two anatomic configurations can be considered to differ in *position* if a 3 degree‐of‐freedom (3DOF) translational correction would adequately align the anatomy as represented in two image sets or associated structure contours. Similarly, two anatomic configurations may differ in *orientation* if the anatomy would be aligned through a 3DOF rotational correction about its center. A difference in both *position* and *orientation* would therefore require a 6 degree‐of‐freedom (6DOF) correction. As 6DOF is sufficient to correct for any rigid‐body difference, residual differences that persist after an optimal 6DOF correction can be attributed to differences in *shape*. Because translational and/or rotational differences can be corrected with 3DOF or 6DOF couch motion typical of standard image‐guidance radiotherapy (IGRT), it is the residual differences in *shape* varying on a daily basis that warrant the new functionality afforded by online ART. Online ART is not limited to accounting for differences in *shape* and can separately or simultaneously be used to correct for variations in *position* and *orientation*. However, as these differences can also be corrected with IGRT, they may not justify the costs associated with online ART in the absence of additional changes in *shape*.

Alternatives to online ART replanning that can account for some changes in *shape* have been described. Notably, selecting a plan from an existing plan library is an effective means when various modes of anatomic change or recurring anatomic configurations are observed.^23^, ^24^, ^25^, ^26^, ^27^, ^28^, ^29^, ^30^, ^31^, ^32^, ^33^, ^34^, ^35^, ^36^, ^37^, ^38^, ^39^ These plan‐of‐the‐day (POD) techniques have the benefit of being tailored (to a degree) to the anatomy of each fraction but without requiring the replanning effort necessitated by current online ART workflows. As a result, the POD technique may represent a strategic approach through which to achieve a portion of the dosimetric benefit of online adaptation without the acute demands on clinical resources that will adversely scale with the benefit. The role of such techniques in relation to newly available daily online ART workflows should be considered in order to inform the strategic allocation of clinical resources while maximizing the clinical benefit to patients.

The purpose of this work was to evaluate the strategic implementation of POD‐based ART schemas using adaptive plans from initial treatment fractions in order to achieve dosimetric benefits similar to daily online ART but without the corresponding cost in clinical resources. We analyzed these schemas with retrospective analysis of our clinical prostate cancer patients treated with CBCT‐guided, daily online ART.

## 2 METHODS

### 2.1 Patients

Evaluation of several POD‐based ART techniques was conducted retrospectively on a cohort of eight patients that received 70.2 Gy in 26 fractions to the prostate and proximal seminal vesicles using CBCT‐guided, daily online ART with the Varian Ethos Treatment platform (Varian Medical Systems, Palo Alto, CA). The vast majority of all target contouring and plan selection tasks conducted during treatment planning and adaptive delivery were overseen by one of two individual radiation oncologists. Clinically, these patients received the adaptive plan in 204 of 208 treatment fractions (98%). Patients were instructed to arrive at treatment with a full bladder and empty rectum.

### 2.2 ART schemas

For each patient, the dose resulting from five different patient alignment and plan creation techniques were compared on individual treatment fractions (see Table 1). The first technique is to utilize the full adaptive capability of the Ethos system to reoptimize a new plan specific to the individual treatment fraction. The plan delivered using this technique is denoted here as the Adapted Plan, or **ADP**.

**TABLE 1:** ART schema characteristics.

	Scheduled plan: Original reference plan	Plan‐of‐the‐day: Most geometrically‐similar plan selected from plan library	Adapted plan: Fully reoptimized daily plan
3 Degree‐of‐freedom patient alignment	SCH‐3DOF	POD‐3DOF	
6 Degree‐of‐freedom patient alignment	SCH‐6DOF	POD‐6DOF	
Patient alignment not required			ADP

The second technique is to deliver the original reference plan based on the simulation CT after 3DOF translational alignment of the patient to correct for differences in position. This is an example of non‐adaptive IGRT and is also clinically available on the Ethos system. The plan delivered using this technique is denoted here as the Scheduled Plan under 3DOF alignment, or **SCH‐3DOF**.

The third technique is similar to SCH‐3DOF in that it features the delivery of the original reference plan but differs in that 6DOF translational and rotational alignment of the patient is used to correct for differences in both position and orientation. The plan delivered using this technique is denoted here as the Scheduled Plan under 6DOF alignment, or **SCH‐6DOF**. Unlike SCH‐3DOF, this technique is not currently a clinically available option on the Ethos system.

The fourth and fifth techniques utilize a POD strategy to deliver an existing plan selected from a previously compiled plan library. Descriptions of the plan library and the selection process are presented below. The selected plan is delivered following either 3DOF or 6DOF alignment of the patient, and the plans delivered using these techniques are denoted here as **POD‐3DOF** and **POD‐6DOF**, respectively.

### 2.3 Image registration

To evaluate and compare the various techniques described above, all available imaging and treatment plan information from each completed treatment fraction was exported from the Ethos Treatment Planning System and imported into the Aria Record and Verify System (Varian Medical Systems, Palo Alto, CA). This included the original reference treatment plan and structure set, along with the CBCT, synthetic CT, and structure set generated during each treatment fraction. All data was available whether or not the adapted plan had been clinically delivered to the patient. In the Aria Image Registration workspace, each fraction's synthetic CT was registered twice to the simulation CT, once using 3DOF (translations only) and once using 6DOF (translations and rotations). These registrations were conducted manually with the qualitative goal of maximizing the overlap of the clinical target volumes contours (CTVs) as depicted on the two image sets.

### 2.4 Plan library creation

**FIGURE 1:**
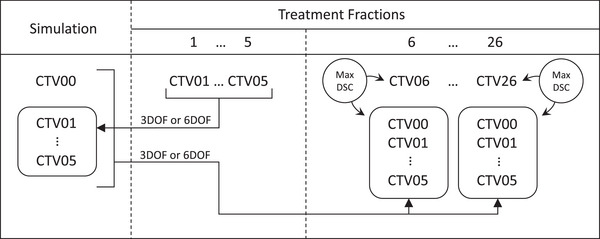
Schematic of the plan library creation and plan selection processes. CTV## denotes the clinical target volume originating from the treatment fraction indicated by ##. *Max DSC* refers to the identification of the maximum Dice similarity coefficient.

For both the POD‐3DOF and POD‐6DOF techniques, a plan library was created composed of the reference plan and the adapted plans from the first five treatment fractions. To compile the plan library and prepare for plan selection, the daily CTV from each of these fractions—which had been reviewed, edited, and approved by the covering radiation oncologist—was propagated using both the 3DOF and 6DOF registrations onto the simulation CT and from there onto the synthetic CTs of the remaining fractions along with the CTV from the simulation CT (see Figure 1). As a result, the synthetic CT for each treatment fraction had 13 CTV structures: one native to that fraction, two propagated from the original simulation CT structure set with 3DOF or 6DOF, and 10 propagated from the first five fractions through the simulation CT with 3DOF or 6DOF.

### 2.5 Plan selection

To select a plan from the plan library that was most appropriate for a given treatment fraction, the Boolean intersection of each propagated CTV and the fraction's native CTV was created and, along with their volumes, used to calculate the Dice similarity coefficient (DSC) for the pair. The plan in the plan library associated with the CTV that yielded the largest DSC was then selected as it was therefore considered to be the most geometrically similar. As an initial exploration into the schemas described here, this work focused on the target volumes alone as these represented the most consistent set of contours and a primary focus for the therapeutic goals of treatment. The registration of each target was conducted manually and independently. Because if this, and because the 3DOF solution space is a subset of the 6DOF solution space, if the 3DOF registration happened to result in a larger DSC than the 6DOF registration, the former was also utilized for the SCH‐6DOF and POD‐6DOF schemas. This remains a valid 6DOF registration solution, simply one with rotations set to 0°. It represents a registration that was, in fact, preferred according to the specific metric of interest (DSC), but which was not originally identified due to the manual processes available for this initial work in existing systems.

### 2.6 Plan transfer, dose recalculation, and dose value evaluation

For each treatment fraction, the plan selected from the plan library was transferred to the synthetic CT via the 3DOF or 6DOF registration and the dose was recalculated. The doses of the SCH‐3DOF and ADP plans, which already existed on the synthetic CTs when transferred from the Ethos Treatment Planning System, were also recalculated to ensure equivalence in dose calculation algorithm and dose grid resolution (AcurosXB v16.1 with 2.5 mm in‐plane dose grid resolution). The original reference plan was also transferred to the synthetic CT via the 6DOF registration and recalculated to provide the SCH‐6DOF technique (except when the 3DOF registration provided a superior DSC as mentioned above). An in‐house software script was then used to export several dosimetric values pertaining to target coverage and organ‐at‐risk (OAR) sparing. Target coverage dose values included the near minimum dose to the CTV (CTV D99%), the volume of the CTV getting the prescription dose (CTV V100%), the near minimum dose to the planning target volume (PTV D99%), and the dose to 95% of the PTV (PTV D95%). OAR dose metric values included the dose to the maximum 2cc of the bowel (Bowel D2cc) and the volume of the bladder and rectum receiving 40 Gy (Bladder V40Gy and Rectum V40Gy).

### 2.7 Analysis

Each target coverage metric value calculated according to the SCH‐3DOF, SCH‐6DOF, POD‐3DOF, or POD‐6DOF technique was normalized to the corresponding ADP value. In this way, the value was interpreted as a proportion of that achieved by the ADP using full reoptimization on a daily basis that was presumed to be optimal. OAR dose metric values were also compared to ADP values, however, in these cases, it was the numerical difference from the ADP value that was used rather than the relative proportion. This was due to the fact that OAR doses tend to vary more between plans than target doses do, as the latter are directed by the prescription dose and normalization. In addition, the values were not necessarily near clinically significant dose objectives and in some cases were very small. Normalizing to these values could produce large relative changes that may not actually be closely associated with doses of clinical consequence. Data regarding the variations in target and OAR dose metric values observed for each ART schema were compared with box‐and‐whisker plots. To statistically compare the four non‐ADP schemas, a Friedman's test was performed followed by post‐hoc Dunn's tests corrected for multiple comparisons within each patient (significant at *p* ≤ 0.05).

The cost in reoptimization effort was determined as the number of treatment fractions where the reoptimization and recalculation of an adapted plan was required. Delivery of the original reference scan required 0 fractions of reoptimization effort, while daily online ART required 26 fractions of reoptimization effort. In comparison, the POD‐based ART schemas featuring a plan library composed of the plans from the initial week of treatment required 5 fractions of reoptimization effort. This work focused on the reoptimization effort alone and did not include the effort associated with additional steps such as recontouring. While these steps are critical,^20^ details of their implementation and the performance of existing systems are likely to evolve quickly. Implementation of POD schemas, too, may vary. By focusing on the reoptimization effort specifically, this work removed the quality effects of the contouring variable in order to directly assess the dosimetric potential of POD schemas to approximate the daily online reoptimization.

## 3 RESULTS

The amount of online reoptimization effort required by the POD schemas was less than 20% of that of the ADP schema (5 vs. 26 fractions). However, this difference was not mirrored proportionally in dose metric values.

**FIGURE 2:**
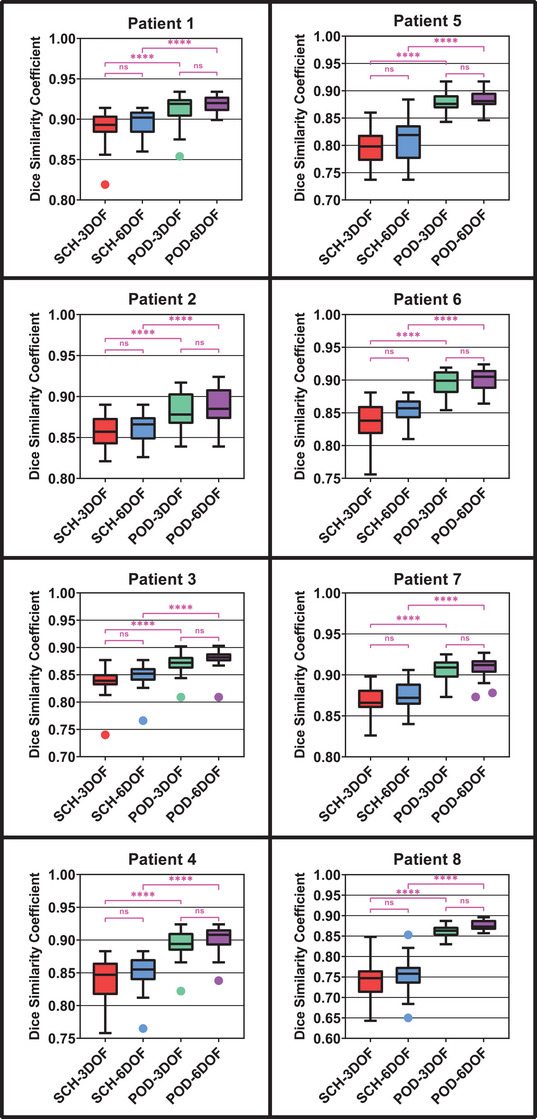
Dice similarity coefficient (DSC) values between the clinical target volumes of given treatment fractions and those of the plans selected for those fractions according to the four non‐ADP schemas. Box limits represent the 25th and 75th quartile, and whiskers were calculated using the Tukey method. Statistical comparisons are denoted as not significant (ns) or significant at *p* ≤ 0.05 (*), *p* ≤ 0.01 (**), *p* ≤ 0.001 (***) or *p* < 0.0001 (****).

Figure 2 depicts the values of the DSC between the CTV of each treatment fraction and that of the plan selected for that fraction according to each non‐ADP schema for all eight patients. In each case, the 6DOF registration did not provide a statistically significant improvement compared to the 3DOF registration. Meanwhile, the use of the POD functionality was associated with an improvement in DSC value that was very statistically significant with *p* ≤ 0.0001.

The difference in dose metrics values across ART schemas was observed to depend on both the individual patient and on the particular dose metric being investigated. For target coverage metrics, the primary effect was that observed between patients. Some patients (e.g., Patient 1 and Patient 4) did not show as much variation in anatomy and dose metric values as others did (e.g., Patient 5 and Patient 8). For patients that did exhibit considerable variation, the POD schemas were observed to approximate the dose metric values of the ADP schema more closely than the SCH schemas did. Meanwhile the 6DOF versions of the SCH and POD schemas didn't result in values much closer to the ADP value than the 3DOF versions.

**FIGURE 3:**
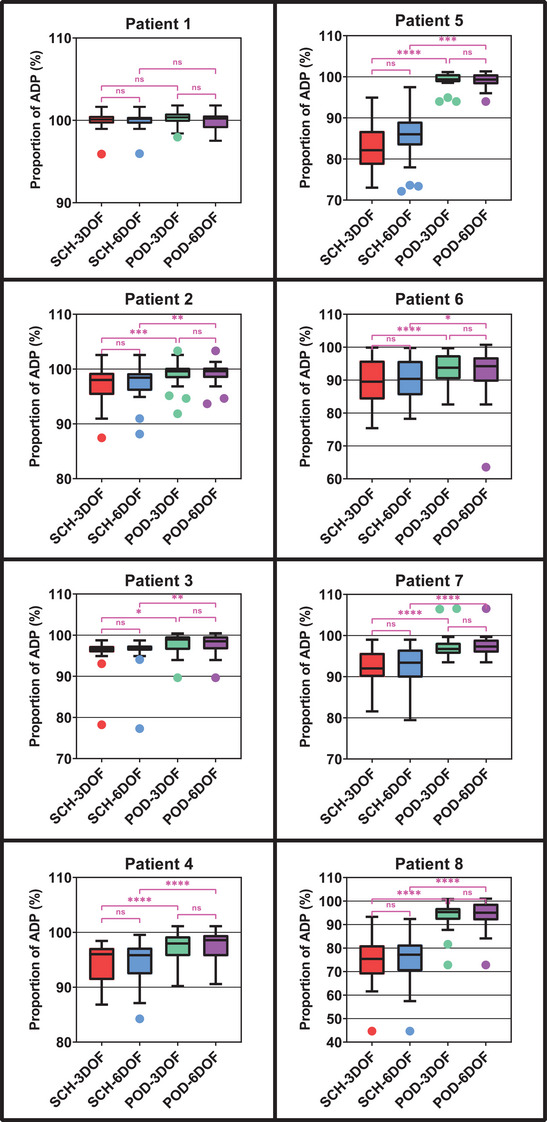
Planning target volume (PTV) D95% dose metric values for the four non‐ADP schemas relative to the values for the ADP schema (100%) for all eight patients. Box limits represent the 25th and 75th quartile, and whiskers were calculated using the Tukey method. Statistical comparisons are denoted as not significant (ns) or significant at *p* ≤ 0.05 (*), *p* ≤ 0.01 (**), *p* ≤ 0.001 (***) or *p* < 0.0001 (****).

For two example patients that did not exhibit much variation, the median D95% for the SCH‐3DOF, SCH‐6DOF, POD‐3DOF, and POD‐6DOF schemas relative to ADP values of 100% were, respectively, 100.1%, 100.2%, 100.4%, and 100.2% (Patient 1) and 96.0%, 95.8%, 98.0%, and 98.6% (Patient 4). For two example patients that did exhibit considerable variation, the values were 82.1%, 86.0%, 99.4%, and 99.3% (Patient 5) and 75.4%, 77.2%, 95.3%, and 95.1% for (Patient 8). Figure 3 depicts the distribution of PTV D95% values for each schema for all eight patients along with the results of the statistical analysis. Descriptive statistics are presented in the table in the Supplemental materials and can be used to calculate the corresponding interquartile ranges.

Similar to those of target coverage metrics, the values of OAR dose metrics also varied considerably patient to patient. However, OAR dose metric values did not exhibit such a consistent dependence on the particular schema.

For two example patients that did not exhibit much variation, the median difference in Bladder V40Gy for the SCH‐3DOF, SCH‐6DOF, POD‐3DOF, and POD‐6DOF schemas from the ADP values were, respectively, 2.6%, 3.2%, 10.2%, and 8.8% (Patient 1) and −1.7%, −3.3%, −3.6%, and −3.4% (Patient 4). For two example patients that did exhibit considerable variation in target coverage metrics but not in OAR dose metrics, the values were −0.3%, 0.1%, 0.2%, and 0.8% (Patient 5) and 8.7%, 6.3%, 2.9%, and 0.7% (Patient 8).

**FIGURE 4:**
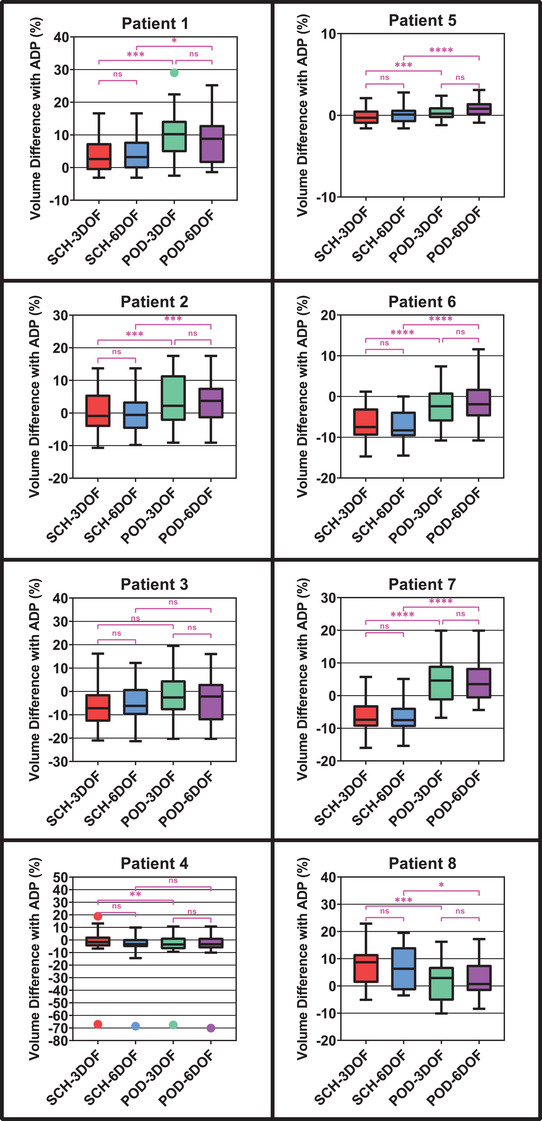
Difference in the Bladder V40Gy dose metric values for the four non‐ADP schemas from the values for the ADP schema for all eight patients. Box limits represent the 25th and 75th quartile, and whiskers were calculated using the Tukey method. Statistical comparisons are denoted as not significant (ns) or significant at *p* ≤ 0.05 (*), *p* ≤ 0.01 (**), *p* ≤ 0.001 (***) or *p* < 0.0001 (****).

For OAR dose metrics, it was also less consistently the case that the fully reoptimized ADP schema value was superior to those from the non‐ADP schemas. These effects may reflect the inconsistency of the OAR dose metric values and their prioritization below those of target coverage metrics, as mentioned in the Discussion. Furthermore, although not prioritized, the OAR dose metric values were generally not observed to be compromised by the POD schemas, either. Figure 4 depicts the distribution of the difference in Bladder V40Gy values of each non‐ADP schema from the ADP value for all eight patients along with the results of the statistical analysis. Descriptive statistics are presented in the table in the Supplemental materials and can be used to calculate the corresponding interquartile ranges.

Additional figures and a table for all remaining metrics and all patients are available in the Supplemental materials.

## 4 DISCUSSION

When retrospectively analyzing patients treated with daily online ART to the prostate and proximal seminal vesicles, we observed that POD‐based ART schemas derived from the plans of the first five fractions were able to achieve the vast majority (typically 80−100%) of the target coverage benefit offered by daily adaptation while requiring less than 20% of the reoptimization effort.

The comparative performance of the SCH‐3DOF, SCH‐6DOF, POD‐3DOF, POD‐6DOF, and ADP schemas was observed to depend on both the individual patient and on the particular dose metric being investigated. Considerable variation was observed among the eight patients studied here. Roughly half of the patients exhibited anatomic variations that manifested in marked differences in the values of dose metrics. For these patients, the ability to deliver a plan tailored to their daily anatomy through some adaptive schema was important for maintaining the desired dose. Meanwhile, dose metric values for the remaining patients were observed to not vary as much across treatments, limiting the clinical benefit offered by online adaptation as compared to the original reference plan. These patient‐specific effects demonstrate that appropriate patient selection remains a lingering challenge of online ART workflows, especially for workflows that are resource‐intensive. Designing workflows that can achieve results similar to those of daily plan reoptimization but with fewer demands on clinical resources could help ameliorate the costs associated with implementing ART when patient selection remains imprecise.

The impact of the various ART schemas also depended on the dose metric being considered, and the patient‐specific effects described above were most apparent for target coverage metrics. The target coverage metrics achieved using the ADP schema were considered the gold standard since the delivered plan was reoptimized to the changing anatomy on a daily basis. Relative to these values, the delivery of the original reference plan (SCH‐3DOF or SCH‐6DOF) frequently reflected considerable compromises in target coverage. However, the POD‐based ART schemas (POD‐3DOF and POD‐6DOF) were able to recover the compromised dosimetry to a large degree (typically 80%−100%), approaching the dose metric values achieved by the ADP schema.

When compared to SCH‐3DOF and POD‐3DOF, the versions of these schemas featuring 6DOF registration provided marginal improvement that was not statistically significant. Because the 3DOF registration solution space is a subset of that of the 6DOF registration, the results from the 6DOF schemas should be at least equivalent to those from the 3DOF schemas. While this was ensured when comparing the DSC value, dose metric values are not equivalent to DSC, which allows for differing behavior between these metrics, including the possibility that a superior DSC value leads to an inferior dose metric value. The limited improvement provided by the 6DOF registration implies that these patients did not demonstrate significant rotational effects separate from changes in shape better addressed using the POD schemas. The functionality of being able to select from a plan library therefore appeared more effective than the ability to conduct 6DOF patient alignment. In addition, it is worth noting that the gold standard ADP dose metric values may be inflated since this technique does not consider the effects of intrafractional motion occurring during the reoptimization process. Therefore, the values achieved by the POD schemas may be even more similar to those actually delivered to the patient.

The behavior of OAR dose metric values, while also patient‐specific, was observed to be less consistent across schemas than that of target coverage metrics. Typically, the values from the SCH and POD schemas reflected compromised OAR sparing as compared to the ADP. However, it was not infrequent that the opposite was true, and better OAR doses were achieved with the non‐ADP schemas. This behavior may in part be due to the fact that our clinical adaptive plan reoptimization normally prioritizes target coverage metrics higher than OAR objectives. In addition, target coverage metrics are more likely to be located near goal values, whereas OAR doses may satisfy or fail to satisfy a dose objective by a sufficient margin so as to not represent an effective avenue for plan improvement. These effects are also sensible considering that OARs were ignored during the image registration and plan selection processes of the POD schemas as implemented here.

Selecting a POD from a plan library is an adaptive strategy that has been of interest for some time. Most often, the technique is applied to treatment sites where anatomic variation is largely governed by a principal factor that influences the position or extent of the target within some range. For example, the position of both the uterus and the bladder are predominantly influenced by bladder filling. Treatment plans can then be generated to correspond to the conditions of a full, empty, or intermediately filled bladder. Furthermore, because multiple levels of bladder filling can be achieved during a single CT simulation appointment, treatment sites like these facilitate the creation of multiple treatment plans upfront, contributing to a plan library available even at the first treatment fraction. The potential and successful implementation of such a strategy for sites like these have been extensively described.^23^, ^24^, ^25^, ^26^, ^27^, ^28^, ^29^, ^31^, ^32^, ^34^, ^35^, ^36^, ^37^, ^39^

The implementation of POD strategies described here differs from the conventional implementation in a number of notable ways. While the position and orientation of the prostate and seminal vesicles are also heavily influenced by bladder filling, they are similarly influenced by variable rectal filling. The interacting effects of these two organs results in a more random set of anatomic configurations. It is therefore less obvious how clinicians are to preemptively subdivide the domain of possible configurations or to conveniently achieve multiple representative instances. The POD schemas described here address this challenge by using the initial week of treatment to sample five instances of possible anatomy with the implicit assumption that this subset will largely be representative of anatomic configurations observed during the remainder of treatment. The fact that our results showed the POD schemas achieve dose metric values approaching those of the ADP schema suggests that this was, in fact, the case, even with as few as five fractions. Furthermore, with this strategy, even during the period where this initial set of plans is being gathered, the dose delivered to the patient is not compromised, as they are receiving fully reoptimized online ART.

In this work, we applied a POD adaptive schema to a treatment site less conventionally considered for the approach and did so by analyzing our experience with real clinical patients treated with CBCT‐guided, daily online ART. By comparing 3DOF and 6DOF versions of both the SCH and POD schemas with the ADP schema, we isolated variables to determine their individual and combined impact on achieving the desired dose metric values. In doing so, we demonstrated that the functionality of selecting a POD from a plan library composed of plans “passively” acquired during an initial week of daily online ART can nearly approximate the dosimetric benefit of a full course of daily online ART but with considerably lower cost in clinical resources. Selecting an existing plan from a plan library may also provide greater flexibility and opportunities for quality assurance as well as compliance with potential reimbursement policies. Our analysis can be helpful for clinicians and vendors alike in determining the best approach to design ART technologies and implement ART workflows to maximize clinical impact in a resource‐conscious way.

Our work is not without its limitations. The eight patients analyzed here represent a relatively small population. However, at the time of analysis, this group was the largest subset of identically treated online ART patients available for study at our institution. Despite the small group size, the consistent benefit of a POD‐based ART schema on target coverage metrics was depicted clearly. However, it remains possible that our observations are not wholly representative of a larger population of similarly treated patients. In addition, many of the observed effects are likely specific to the studied treatment site because the efficacy of a plan library will depend on the details of anatomic variability that is typical for each site.

Another specification of our work is that we used a 1‐week period of daily online ART to generate plans for the plan library. This duration was ultimately a balance between accumulating a sufficient sample of anatomic configurations in the plan library while also limiting the number of treatments requiring online reoptimization. This choice was informed by prior analysis conducted internally at our institution that suggested the anatomic variations governing the shape and position of the target as observed during the initial week of treatment for these patients was largely indicative of the remainder of their course of treatment. Different treatment sites, fractionation schemes, or even individual patients may demand longer or shorter durations for this initial period. Of course, if a novel anatomic configuration that is not represented in the plan library is observed, the POD schemas may not have an appropriate plan available. In these cases, the patient setup could be adjusted or online reoptimization, if available, could be implemented. In the latter case, the new plan could be subsequently added to the plan library for future treatments. A model of anatomic variation could also be generated from examples of daily anatomy and additional plans could be created through interpolation and extrapolation. Our work did not include any such model but was limited to a one‐to‐one “bootstrapping” selection from six specific plans (the original reference plan and those from the five initial treatment fractions).

Finally, while complete optimization of the POD schemas is beyond the scope of this work, it is worth noting some particular variables that could have an additional effect on its performance. For both POD schemas, image registration was conducted manually with the qualitative objective of maximizing the overlap between the daily CTV and the CTVs of the candidate plans in the library. Using an automatic image registration algorithm may improve speed, reproducibility, and robustness of this process. In addition, including OAR contours such as the bladder and rectum into this process may provide a more comprehensive alignment. Similarly, additional structures could be considered during plan selection, so that the selected plan is more appropriate when considering the target and OARs. Our approach to plan selection also revolved around maximizing the DSC. While this was a straightforward metric that could be calculated quickly, alternative metrics, including dosimetric ones, could be considered and might offer additional advantages. Ultimately, if dose calculation was sufficiently fast, one could imagine calculating dose on either a sample of plans or the entire set of plans within the library to precisely determine the most appropriate POD.

Despite these limitations, our work demonstrates that deliberate consideration of anatomic variations and strategic implementation of adaptive schemas may allow clinicians to achieve the majority of the dosimetric benefits of daily online ART with workflows that are considerably more practical with respect to clinical resources. These strategies may help with several of the current barriers to more widespread utilization of ART such as staffing, quality assurance, and billing concerns, ultimately leading to more adaptation and improved dose distributions delivered to patients. Lastly, schemas like those described here can be combined with others as part of a broader ART strategy, and our analysis can inform clinicians and vendors as to what improvements are most likely to advance the technique.

## 5 CONCLUSION

In this work we demonstrated several ART schemas that approached the dosimetric efficacy of daily online ART for patients treated to the prostate and proximal seminal vesicles yet required a small fraction of the online reoptimization effort. Using the non‐linear relationship between the dosimetric benefit and the clinical resource cost of ART along with the appropriate implementation of various workflows may be critical for promoting its practical implementation and for maximizing patient benefit.

## AUTHOR CONTRIBUTIONS

A.D.Y. contributed to the conception and design of the work, data acquisition, data analysis and interpretation, and creation and review of the manuscript. A.C. contributed to the conception and design of the work, data acquisition, data analysis and interpretation, and creation and review of the manuscript. M.M. contributed to the data acquisition and creation and review of the manuscript. E.S. contributed to the conception and design of the work, data analysis and interpretation, and creation and review of the manuscript. K.H. contributed to the data acquisition and creation and review of the manuscript.

## CONFLICT OF INTEREST STATEMENT

A.D.Y. reports a patent regarding an adaptive radiotherapy phantom.

## Supporting information

Supporting Information

## Data Availability

Research data are not shared.
