# Supplementary material for: Empirical analysis of a plan‐of‐the‐day strategy to approximate daily online reoptimization for prostate CBCT‐guided adaptive radiotherapy
Source: J Appl Clin Med Phys. 2023 Nov 29;25(1):e14221. doi: 10.1002/acm2.14221 (PMC10795443; doi:10.1002/acm2.14221)
Supplement: Supplementary file 1 — Supporting Information [file ACM2-25-e14221-s001.docx]

**SUPPLEMENTAL MATERIALS**

PTV D99%

Supplemental Fig. 1: Planning Target Volume (PTV) D99% dose metric values for the four non-ADP schemas relative to the values for the ADP schema (100%) for all eight patients. Box limits represent the 25^th^ and 75^th^ quartile, and whiskers were calculated using the Tukey method. Statistical comparisons are denoted as not significant (ns) or significant at p ≤ 0.05 (*), p ≤ 0.01 (**), p ≤ 0.001 (***) or p < 0.0001 (****).

CTV D99%

Supplemental Fig. 2: Clinical Target Volume (CTV) D99% dose metric values for the four non-ADP schemas relative to the values for the ADP schema (100%) for all eight patients. Box limits represent the 25^th^ and 75^th^ quartile, and whiskers were calculated using the Tukey method. Statistical comparisons are denoted as not significant (ns) or significant at p ≤ 0.05 (*), p ≤ 0.01 (**), p ≤ 0.001 (***) or p < 0.0001 (****).

CTV V100%

Supplemental Fig. 3: Clinical Target Volume (CTV) V100% dose metric values for the four non-ADP schemas relative to the values for the ADP schema (100%) for all eight patients. Box limits represent the 25^th^ and 75^th^ quartile, and whiskers were calculated using the Tukey method. Statistical comparisons are denoted as not significant (ns) or significant at p ≤ 0.05 (*), p ≤ 0.01 (**), p ≤ 0.001 (***) or p < 0.0001 (****).

Bowel D2cc

Supplemental Fig. 4: Difference in the Bowel D2cc dose metric values for the four non-ADP schemas from the values for the ADP schema for all eight patients. Box limits represent the 25^th^ and 75^th^ quartile, and whiskers were calculated using the Tukey method. Statistical comparisons are denoted as not significant (ns) or significant at p ≤ 0.05 (*), p ≤ 0.01 (**), p ≤ 0.001 (***) or p < 0.0001 (****).

Rectum V40Gy

Supplemental Fig. 5: Difference in the Rectum V40Gy dose metric values for the four non-ADP schemas from the values for the ADP schema for all eight patients. Box limits represent the 25^th^ and 75^th^ quartile, and whiskers were calculated using the Tukey method. Statistical comparisons are denoted as not significant (ns) or significant at p ≤ 0.05 (*), p ≤ 0.01 (**), p ≤ 0.001 (***) or p < 0.0001 (****).

Supplemental Table: Descriptive statistics of the difference in all dose metric values for the four non-ADP schemas from the values for the ADP schema for all eight patients. (A) SCH-3DOF, (B) SCH-6DOF, (C) POD-3DOF, (D) POD-6DOF.
